# Effects of 462 nm Light-Emitting Diode on the Inactivation of *Escherichia coli* and a Multidrug-Resistant by Tetracycline Photoreaction

**DOI:** 10.3390/jcm7090278

**Published:** 2018-09-12

**Authors:** Shiuh-Tsuen Huang, Chun-Yi Wu, Nan‐Yao Lee, Chien-Wei Cheng, Meei-Ju Yang, Yi-An Hung, Tak-Wah Wong, Ji-Yuan Liang

**Affiliations:** 1 Department of Science Education and Application, National Taichung University of Education, Taichung 40306, Taiwan; hstsuen@mail.ntcu.edu.tw; 2 Department of Biotechnology, Ming-Chuan University, Gui-Shan 33343, Taiwan; kevin1060755@gmail.com (C.-Y.W.); ochien@gmail.com (C.-W.C); amyhung840809@gmail.com (Y.-A.H.); 3 Division of Infection, Department of Internal Medicine, National Cheng Kung University Hospital, Department of Medicine, College of Medicine, National Cheng Kung University, Tainan 70101, Taiwan; nanyao@mail.ncku.edu.tw; 4 Tea Research and Extension Station, Taoyuan 32654, Taiwan; 762204@gmail.com; 5 Department of Dermatology, National Cheng Kung University Hospital, Department of Biochemistry and Molecular Biology, College of Medicine, Center of Applied Nanomedicine, National Cheng Kung University, Tainan 70101, Taiwan

**Keywords:** blue light, inactivation, *MDR E. coli*, skin and soft tissue infections (SSTIs), tetracycline

## Abstract

The adaptability of bacterial resistance to antibiotics contributes to its high efficiency during evolution. Tetracycline (TC) is a broad-spectrum antimicrobial agent. Chromatographic analyses and mass spectrometry were used to study the effects of the light illumination of a 462 nm light-emitting diode (LED) on the conformational changes of TC in a phosphate buffer solution (PBS, pH 7.8). Especially, the inactivation of superoxide anion radicals (O_2_•^−^) and *Escherichia coli* (*E. coli*), including that of a multidrug-resistant *E. coli* (*MDR E. coli*), were investigated during the photolysis of TC. A photolysis product of TC (PPT) was generated in an alkaline solution after the illumination of a blue light. The mass spectra of PPT had characteristic ion signals in m/z 459, 445, and 249.1 Da. The PPT has the molecular formula of C_22_H_22_N_2_O_9_, and the exact mass is 458.44 g/mol. The inactivation of *MDR E. coli* is not significant with TC treatment. The drug-resistant ability of *MDR E. coli* has a less significant effect on PPT, and the changed conformation of TC retained the inactivation ability of *MDR E. coli* upon blue light photoreaction. With TC, illuminated by a blue light in a pH 7.8 PBS, O_2_•^−^ was generated from TC photolysis, which enhanced the inactivation of *E. coli* and *MDR E. coli*. A 96.6% inactivation rate of *MDR E. coli* was reached with TC under 2.0 mW/cm^2^ blue light illumination at 25 ± 3 °C for 120 min, and the effects of the TC-treated photoreaction on *MDR E. coli* viability repressed the growth of *MDR E. coli* by 4 to 5 logs. The present study of the blue light photoreaction of TC offers a new approach to the inactivation of *MDR E. coli.*

## 1. Introduction

Skin and soft tissue infections (SSTIs) comprise a wide spectrum of clinical presentations, ranging from simple cellulitis to life-threatening necrotizing fasciitis. SSTIs account for around 10% of annual hospital admission in the USA [1]. Aerobic Gram-positive *Staphylococcus aureus* (*S. aureus*) and β-haemolytic streptococci are frequently the etiology of acute SSTIs, while aerobic Gram-negative bacilli and anaerobes are most commonly isolated from immunocompromised hosts or patients with decreased vascular perfusion, such as diabetic foot infections [2]. In 2012, the Assessing Worldwide Antimicrobial Resistance Evaluation (AWARE) surveillance program evaluated isolates from 163 US medical centers and reported that *S. aureus* (55.5%), *Escherichia coli* (5.9%), and *Klebsiella spp*. (5.5%) are the most frequently identified bacteria [2]. More importantly, resistant Gram-negative pathogens, such as extended-spectrum β-lactamases (ESBLs) *Escherichia coli* (*E. coli*), increased to 9% in 6 years [2]. 

In addition to SSTIs, *E. coli* is a Gram-negative bacterium commonly found in industrial environments and animal digestive systems. *E. coli* is also a microorganism that indicates the existence of pathogenic microorganisms in the testing environment [3]. Many strains of *E. coli* are also important human pathogens, which produce various toxins accompanied with various syndromes, such as gastroenteritis, urinary tract infection, meningitis, peritonitis, and septicemia [4,5]. 

The TC molecule contains a linear fused tetracyclic nucleus, to which a variety of functional groups are attached. TC is a broad-spectrum and highly effective antibiotic material. TC represses bacteria by inhibiting protein synthesis through the prevention of the association of aminoacyl-tRNA with the bacterial ribosome [6].

TC is also widely used in the agriculture industry to prevent animal disease and as a subtherapeutic growth promoter [7]. TC resistance has been the most common type of resistance observed in animal isolates since its discovery in 1948 [4,8]. After systemic administration, more than 70% of the unchanged form of TC is excreted from humans and animals by means of urine and feces into the environment. Waste containing TC poses a serious threat to ecosystems [9]. TC was reported to be one of the highest antimicrobial resistances of *E. coli* during evolution [4]. Furthermore, a toxin-producing *E. coli* that can be transmitted to humans has been discovered in animals [10]. Antimicrobial resistance is a major global health threat in both human and veterinary medicine [11]. The new antibiotic development process usually takes decades. Therefore, a new strategy to overcome drug-resistant *E. coli* is a healthcare emergency.

Reactive oxygen species (ROS), including hydrogen peroxide (H_2_O_2_), the hydroxyl radical (•OH), the peroxyl radical (ROO•), and the superoxide anion radical (O_2_•^−^), are generally reactive molecules or radical species [12]. It has been reported that riboflavin treated with blue light illumination can be applied to inactivate *E. coli*, *S. aureus*, and methicillin-resistant *S. aureus* (MRSA) by DNA cleavages, caused by O_2_•^−^ generation [3,13,14]. O_2_•^−^ is an intermediate, produced via oxidation–reduction reactions. The oxidative stresses can cause tissue damage, inflammation, and atherosclerosis in aging cells [15,16].

Phototoxicity is a well-known side effect in patients taking tetracycline. The phototoxicity of TC is partially oxygen-dependent, possibly due to its involvement with the singlet oxygen [17]. The degradation of TC can occur when TC is treated with ultraviolet UV light illumination. TC absorbs UVA light and is considered light-sensitive and unstable in water [18]. In another study, ROS was generated via the photodegradation of TC treated with simulated sunlight illumination [19]. Photochemical reactions under solar illumination are considered one of the most important ways to enable antibiotic degradation in natural aquatic environments [20]. It has been reported that the majority of TCs are transformed into intermediate products without complete mineralization under TC photolysis. The photolytic product of TC (PPT) brought a higher adversity risk to bacteria during UV photolysis [21]. The conformational changes of TC may change their functional characteristics and affect the inactivation of microorganisms under photochemical reactions. However, UV light makes up 8–9%, while the visible light represents 46–47%, of the total energy of the sun. It would be of interest to further investigate whether the photosensitization of TC can occur via visible light photoreaction, and whether ROS can be generated from TC photodegradation via visible light illumination. 

The adaptability of bacterial resistance to antibiotics comes from its high efficiency evolutionary mutation rate, and new methods were developed for clinical usage to solve this difficult situation. One of the novel approaches is the antibacterial photodynamic inactivation of bacteria (aPDI) in order to achieve further effective action against available bacteria [22]. The key advantage of the local aPDI treatment is independent of the bacterium resistance pattern. The aPDI treatment has become a potential alternative or adjuvant in treating SSRIs [23,24], and it is used to decrease nosocomial infections of the skin with multiresistant bacteria [25]. The application of aPDI to *E. coli* with tetracyclines, such as demeclocycline [26,27], doxycycline [26,27], chlortetracycline [28], oxytetracycline [27], and tetracycline [26,29], requires a UV light for the inactivation of *E. coli*. Demeclocycline is a semisynthetic tetracycline. It has been reported that demeclocycline can be excited by violet light (415 nm) and UVA light (365 nm), and it is able to eradicate Gram-positive (MRSA) and Gram-negative (*E. coli*) bacteria [26]. Lights with shorter wavelengths and high energy may cause damage to cells. Further research could investigate whether the ROS is due to TC photolysis and the PPT enhances the adversity risk to bacteria, including a resistance microbial via blue light illumination.

TC under visible light illumination treatment could be potentially degraded. Little is known about this photodegraded product. This study inspects the TC photodegraded products after blue light illumination. The main purpose of this study is to examine the effects of aPDI on *E. coli* and *MDR E. coli* by the irradiation of TC with a blue LED light (462 nm). The production of O_2_•^−^ from light-excited TC and structural changes of TC under blue light illumination were also examined. The effects of TC under blue light illumination on microbial viability are used as an indicator of the character of the hygienic environment in order to measure the efficiency of the technique. 

## 2. Materials and Methods

### 2.1. Organization of the Photoreaction System

**Figure 1:**
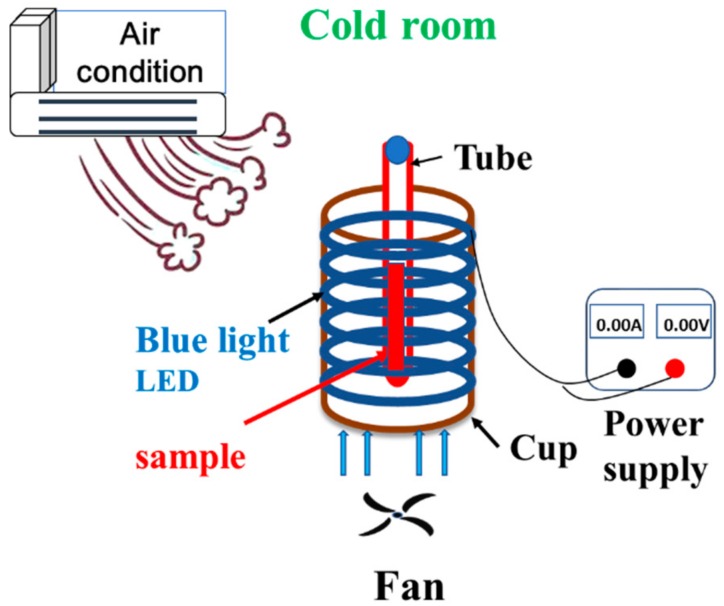
Organization of the photoreaction system.

The photoreaction system was formed in a nontransparent plastic cup (height: 8 cm; diameter: 7 cm), equipped with a light-emitting diode (LED) lamp [30]. The reaction samples were put in a glass test tube and placed to the top of a plastic column, as shown in Figure 1. In this study, six blue, green, and red LED lamps (DC 12 V 5050, vita LED Technologies Co., Tainan, Taiwan) were used as lighting sources, and the LED lamps were placed in the white cup. Blue, green, and red LED lamps emitted maxima wavelengths of 462, 529, and 632 nm, respectively. The spectral widths at half the height (W_1/2_) were 23, 31, and 14, respectively. The power supply (YP30-3-2, Chinatech Co., New Taipei City, Taiwan) was used to monitor light irradiance, and the irradiation (mW/cm^2^) was inspected with a solar power meter (TM-207, Tenmars Electronics Co., Taipei, Taiwan). During the illumination process, the photoreaction system was put in a cold room at 10 ± 1 °C, along with an infrared thermometer (MT 4, Raytek Co., Santa Cruz, CA, USA) to maintain the low temperature.

### 2.2. Chemicals

**Figure 2:**
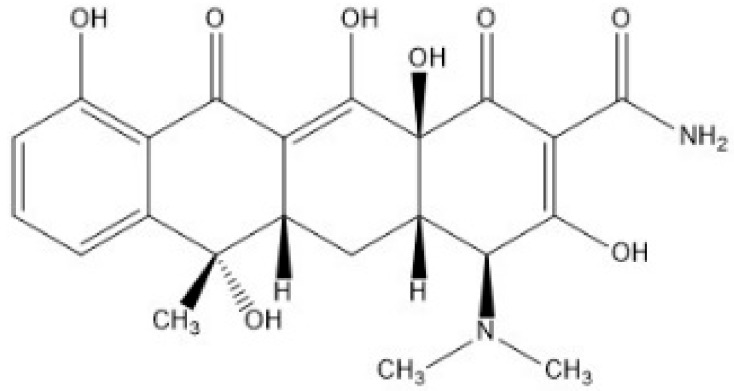
The chemical structure of tetracycline.

Tetracycline (TC) has a linear fused tetracyclic nucleus, as shown in Figure 2. The chemicals, such as L-methionine, mono-potassium phosphate, potassium di-hydrogen phosphate, and tetracycline, were all bought from Sigma-Aldrich Co. (St. Louis, MO, USA). Nitro blue tetrazolium chloride (NBT) was bought from Bio Basic Inc. (Markham, Ontario, Canada). Ultrapure water, purified by a Milli-Q system, was used as a solvent throughout this study.

### 2.3. Effects of Colour Lights on TC Photolysis

The effects of colour lights on TC photoreaction were studied by a UV/Vis spectrometer. In brief, (A) a 50 mg/L TC in 0.1 M PBS (pH 7.8) in the dark was used as a standard solution, and (B) a 50 mg/L TC in 0.1 M PBS (pH 7.8) were illuminated by blue, green, or red light at 2.0 mW/cm^2^ for 1 to 4 h. The absorbances of the TC solutions were measured at 200–800 nm by a UV/Vis spectrometer (Lambda35, Perkin-Elmer).

### 2.4. LC-MS/MS Analysis of TC Photolysis

The TC and the photolysis product of TC (PPT) were examined using LC-MS/MS analysis, as described in previous studies [21,31]. Briefly, (A) a 50 mg/L TC in 0.1 M PBS (pH 7.8) in the dark was used as a standard, and (B) a 50 mg/L TC in 0.1 M PBS (pH 7.8) was illuminated by blue light at 2.0 mW/cm^2^ for 120 min. The analyses of TC and PPT were studied by a high performance liquid chromatography (HPLC) and a 6410B triple quadrupole mass spectrometer (Agilent 1200 Series, Agilent Technologies, Palo Alto, CA, USA), equipped with an electrospray ionization (ESI) source. TC and PPT were detected in positive-ion mode and under the following conditions, drying gas (N_2_) temperature and flow, 350 °C, and 11 L/min; nebulizer gas pressure, 50 psi; capillary voltage, 4 kV, and capillary temperature, 280 °C. MassHunter workstation software in version B.06.00 was used for the data analysis.

TC and PPT were separated using an Agilent Poroshell 120 EC-C_18_ column (2.7 µm, 4.6 mm id × 150 mm, Agilent Technologies, Palo Alto, CA, USA). The separation process was performed by a mobile phase consisting of solvent A (0.1% formic acid) and solvent B (methanol), with the gradient elution as follows. A mixture of 70% A and 30% B was used during the first 7 min. The linear gradient started with 7–12 min, 70–30% solvent A; 12–14 min, 30–5% solvent A; 14–16 min, 5–5% solvent A; and 16–17 min, 5–70% solvent A. The end of mobile phase was to be 70% solvent A from 17 to 22 min. The sample injection volume was 10 µL, and the flow rate was 500 μL/min.

### 2.5. Detection of O_2_•^−^

Both direct and indirect methods can be utilized to detect active O_2_•^−^. The direct method requires particular apparatus, such as an electron spin resonance (ESR) spectroscopy, while the indirect ways are easily exercised in analysis [12]. The nitro blue tetrazolium (NBT) reduction method is an indirect assay for determining O_2_•^−^, and NBT can be reduced by the O_2_•^−^ from intermediates [32]. The NBT reduction method is an O_2_•^−^-scavenging assay and can detect the quantity of O_2_•^−^ [16,33].

The effect of TC photolysis under blue light illumination on O_2_•^−^ generation was inspected. The NBT reduction method was modified from the photoreaction of riboflavin and FMN, and it acted an index of O_2_•^−^ creation, as described previously [30,32].

All reagents were newly prepared before the tests. First, 109.3 mg of L-methionine was added into a 75 mL 0.1 M PBS (pH 7.8). Then, 10 mg NBT and 25 mL 200 mg/L TC were added into the solution. Finally, the concentrations of TC, methionine, and NBT were 50, 1090, and 100 μg/mL in the reactant, respectively. The solution was treated with blue light illumination at 2.0 mW/cm^2^ for 30, 60, 90, and 120 min at room temperature. O_2_•^−^ was generated from the photochemical reaction, by which NBT was reduced and generated formazan, which can be measured at 560 nm.

### 2.6. Effects of TC on E. coli and MDR E. coli Under Blue Light Illumination (During Illumination)

This study inspected the effects of TC treated with blue light illumination on the inactivation of *E. coli* in a photochemical reaction system. *E. coli*, DH5α strain (NCBI Taxonomy ID: 668369), and a single colony of *MDR E. coli*, clinical isolate from the National Cheng Kung University Hospital (Taxonomy ID: IE005), were grown overnight in LB broth at 37 °C. The *MDR E. coli* IE005 was isolated from from a patient’s wound, not an epidemic strain. All pathogens obtained from NCBI and National Cheng-Kung University Hospital passed through the ethics approval process of the National Cheng-Kung University Hospital Biosafety Committee (Project identification code: 8000-4-00-301).

After evolution, 0.5 mL of *E. coli* was transferred to a 1.5 mL centrifuge tube and diluted with sterilized water. Cultures increased to an optical density of 600 nm (OD_600_) at 0.5 (ca. 4.1 × 10^7^ CFU/mL). 

After 10 min of centrifugation at 10,000 rpm, the supernatant was removed, and 1 mL 0.1 M PBS (pH 7.8) was added. Then, the bacterial solution was diluted with the TC solution (50 mg/L TC in 0.1 M pH 7.8 PBS) and moved to a glass tube, treated with or without blue light illumination. Briefly, (A) a 0.1 M PBS (pH 7.8) in the dark was used as a control, (B) a 50 mg/L TC in 0.1 M PBS (pH 7.8) was kept in the dark, (C) a 0.1 M PBS (pH 7.8) was treated with blue light illumination at 2.0 mW/cm^2^ for 120 min, and (D) a 50 mg/L TC in 0.1 M PBS was treated with blue light illumination at 2.0 mW/cm^2^ for 120 min. For the dark control, thick aluminium foils were used to cover the plate and tubes to avoid blue light illumination. During the illumination, the photoreaction system was put in a cold room and maintained at a temperature of 10 ± 1 °C. The illumination was also conducted at room temperature (25 ± 3 °C) to examine the temperature effects of the aPDI of TC on *MDR E. coli* inactivation. 

After the photoreaction, 0.2 mL reaction solutions with sterilized water were transferred to Luria agar plates and incubated at 37 °C overnight. After treatment, the survival of *E. coli* or *MDR E. coli* was examined by viable plate colony counts. The inactivation rate of *E. coli* or *MDR E. coli* was examined as the percentage of the decrease (= (1 − *T/C*)×100%, where *T* is the number of CFU of the test groups (*T*) and *C* is that of the control in the dark (*C*)). Thus, the reduction percentage is defined as the negative value of the inactivation rate.

### 2.7. Effects of TC Photo-Products on the Viability of E. coli and MDR E. coli (After Illumination)

The effects of PPT on the *E. coli* and *MDR E. coli* viability were inspected, after blue light exposure, at 2.0 mW/cm^2^ for 120 min in the presence of TC. Briefly, (A) a 0.1 M PBS (pH 7.8) in the dark was used as a control, (B) a 50 mg/L TC solution in 0.1 M PBS (pH 7.8) was kept in the dark, and (C) a 50 mg/L TC solution in 0.1 M PBS (pH 7.8) was treated with blue light illumination at 2.0 mW/cm^2^ for 120 min in a cold room (10 ± 1 °C) or kept at room temperature (25 ± 3 °C).

*E. coli* or *MDR E. coli* was incubated in Luria broth at 37 °C overnight. After growing overnight, 0.5 mL of the incubated solution was moved to a 1.5 mL centrifuge tube and diluted with sterilized water. After 10 min of centrifugation at 10,000 rpm, the supernatant was removed. Then, 1 mL of the 0.1 M phosphate buffer was added. After being thoroughly mixed, the bacterial solution was diluted with a solution of TC or PPT and incubated at room temperature for 60 min. Then, 0.2 mL of the reaction solution was moved to a Luria agar plate and incubated at 37 °C overnight. The inactivation rate of *E. coli* or *MDR E. coli* was examined, as described in Section 2.6. 

### 2.8. Statistics

Each independent experiment was performed on every different day, three times for every test. For every single test, each bacterial sample was examined in a specific experimental condition. Data are represented as the mean ± standard deviation of each individual experiment. A homoscedastic two-sample *t*-test was used to evaluate if the two groups of measurements differed. A *p*-value less than 0.05 was considered statistically significant.

## 3. Results

### 3.1. Effect of Different Wavelengths of Visible Light on the Photolysis of TC 

**Figure 3:**
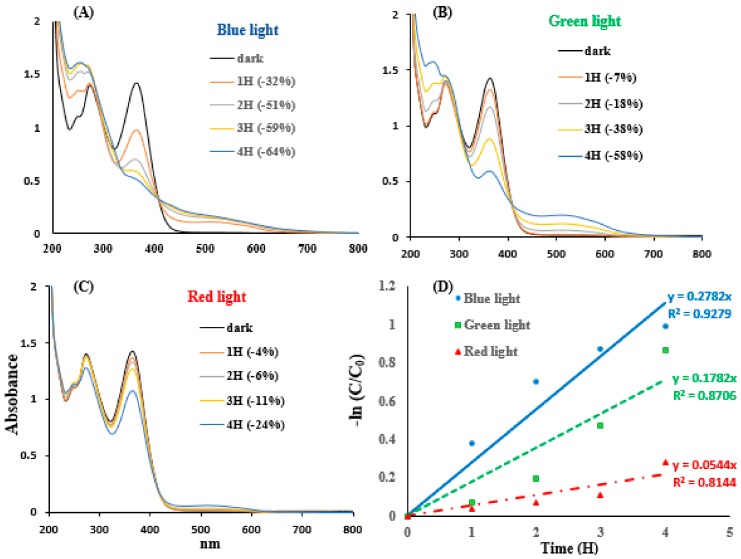
Spectra of 50 mg/L TC illuminated by (**A**) blue, (**B**) green, and (**C**) red light illuminations at 2.0 mW/cm^2^ for 1 to 4 h. In (**D**), the k_appa._ of TC treated with different visible light illuminations.

The photolysis of TC treated with visible light illumination was investigated. TC spectra were studied by blue, green, and red light illumination, as shown in Figure 3A–C, respectively. TC had two absorption bands, at 272 nm and 364 nm, in the dark. The photolysis of TC, under different blue, green, and red light illumination for 1 to 4 h, displayed different absorption bands. The TC absorbance at 364 nm was intensively reduced via blue light illumination. Blue light has the highest efficiency in relation to TC photolysis. The red light had the least influence, because the absorbance at 364 nm was not significant. The apparent degradation rate constants of TC, under different visible light illuminations, were inspected as follows. 

Niu et al. (2013) reported that the TC under solar, UV-A, UV-C, and Xenon lamp light irradiation showed that the light effect on TC photolysis kinetics followed a pseudo-first-order reaction [34]. As shown in Figure 3(D), the absorbance of TC at 364 nm was measured by different visible light illuminations, and the reaction rate of the TC expression is simplified into a pseudo-first-order kinetic model (Equation (1)). Equation (2) can be obtained by integrating Equation (1) with *t* = 0 and *t* = t. Therefore, the kinetics of TC photolysis in a PBS (pH 7.8) was simulated by a pseudo-first-order kinetic model, as follows.

(1) $$\;-\frac{dC_{TC}}{dt}=k_{appa.}\times C_{TC}\;\;\;\;$$

(2) $$\;-\ln〔\frac{C_{TC}}{C_{0}}〕=k_{appa.}\times t\;$$

C_0_ and C*_TC_* are the TC initial concentration and the TC concentration at reaction time t, respectively. *k_appa_*_._ is the apparent decomposition rate constant. As shown in Figure 3D, the plot of -ln(*C*/*C*_0_) versus the reaction time (*t*) yielded straight lines, and the value of the correlation coefficient (*R*^2^) of blue light illumination treatment was higher than 0.92, which indicated that TC photolysis followed a pseudo-first-order kinetic under blue light illumination in this study. As shown in Figure 3D, the values of *k_appa_*_._, under blue, green, and red light illumination at 2.0 mW/cm^2^, were 0.28, 0.18, and 0.054 (h^−1^), respectively. TC photolysis kinetics showed that the TC photolysis efficiency was faster under a blue light condition than under green and red light conditions, and the better property of TC photolysis may be attributed to its photosensitive efficiency, caused by blue light.

### 3.2. Molecule Identification by LC-MS/MS Analysis

**Figure 4:**
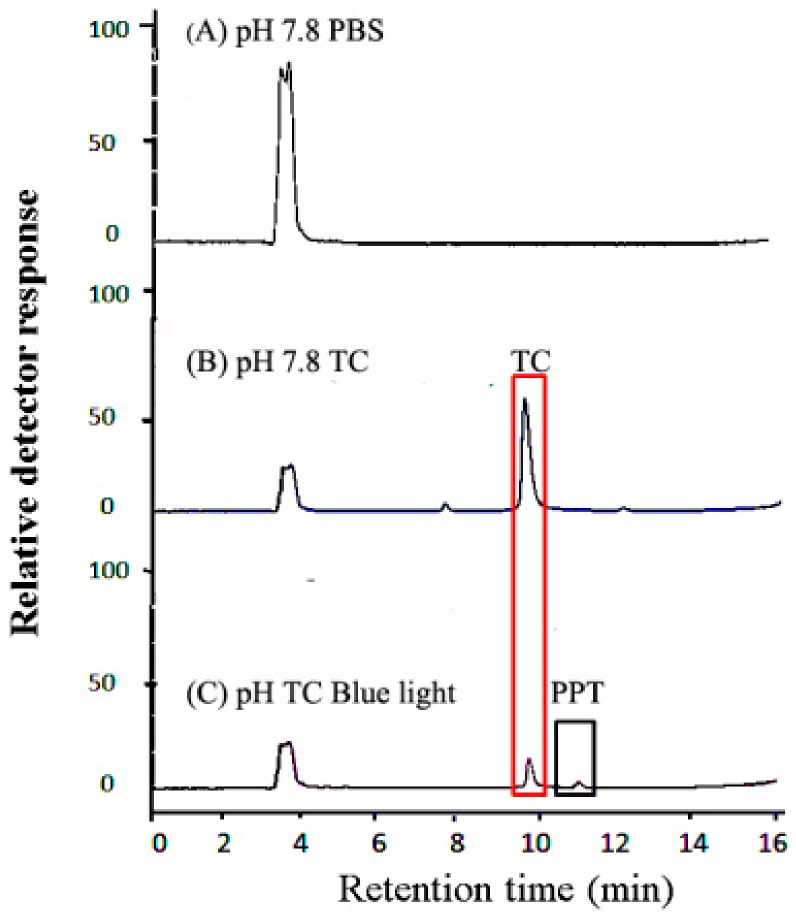

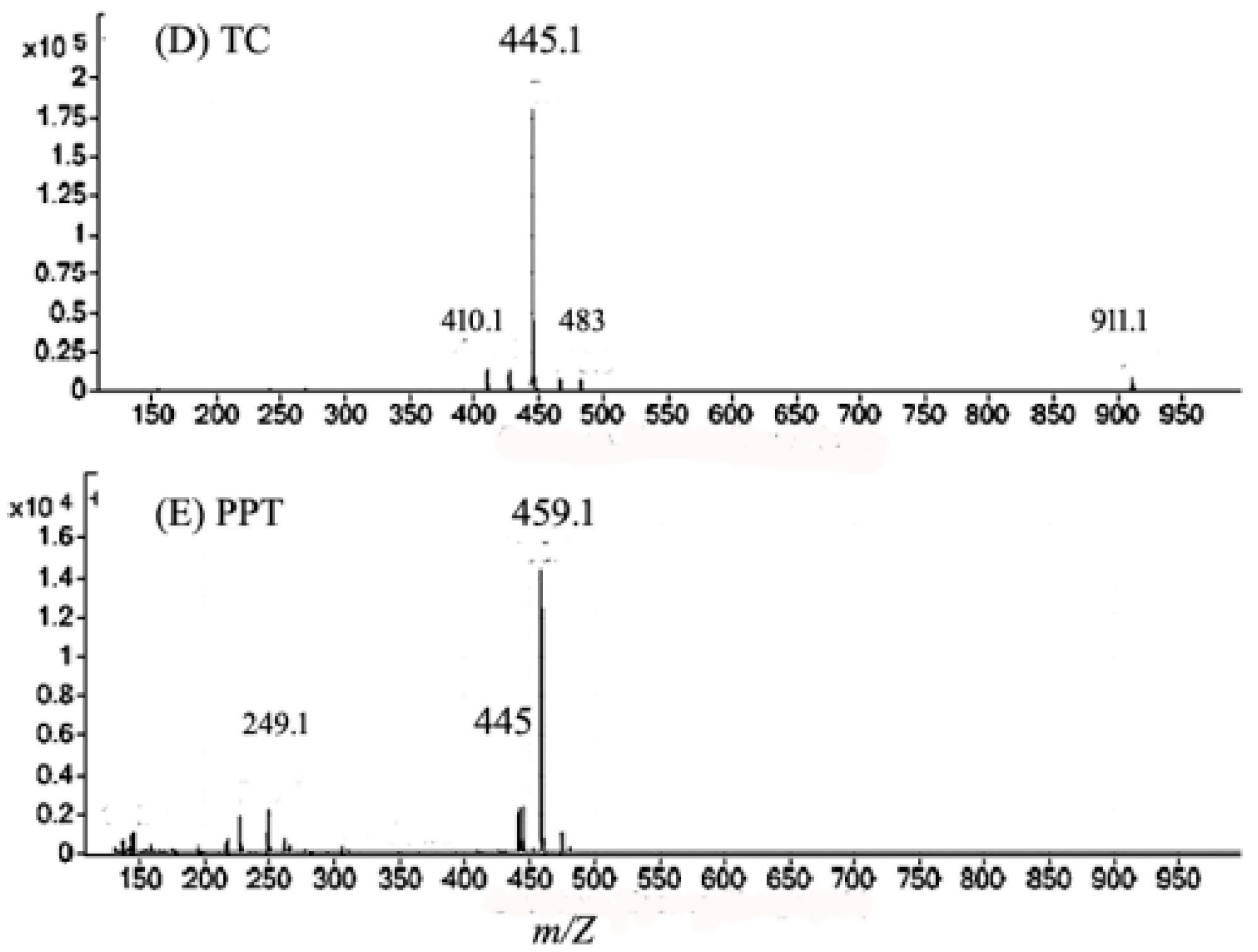
Total ion chromatogram of an HPLC-MS analysis of a (**A**) PBS (pH 7.8), (**B**) 50 mg/L TC solution, and (**C**) TC treated with blue light illumination at 2.0 mW/cm^2^ for 120 min. (**D**) Electron ionization mass spectra of TC, and the photolysis product (E, PPT) from TC treated with blue light illumination for 2 h. Ion spectra product of [M–H]^+^ for a blue-light-treated TC solution (pH 7.8). The selected precursor ion is m/z 445, and the proposed fragments are shown.

Under blue light illumination, the photochemical products were studied by the LC/MS/MS method, and the ion spectra of PPT are shown in Figure 4. As shown in Figure 4A, total ion chromatogram of an HPLC-MS analysis of the PBS (pH 7.8) was observed at 3.5 min. As shown in Figure 4B, TC was observed at 9.8 min, and the major ion fragment is m/z 445.1 Da in Figure 4D. The PPT was observed at 10.3 min after blue light illumination, as shown in Figure 4C. The mass spectrum of PPT had characteristic ion signals in m/z 459.0, 445.0, and 249.1 Da, as shown in Figure 4E.

### 3.3. Probing of Radicals in the Photolysis of TC Under Blue Light Illumination

**Figure 5:**
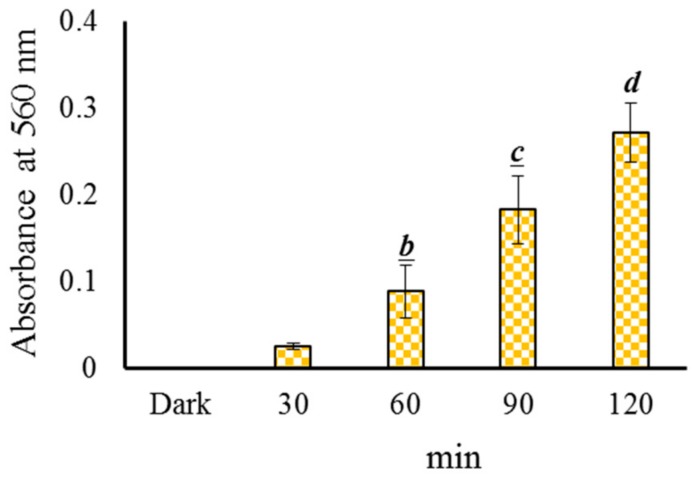
Effects of 50 μg/mL TC on NBT reduction by blue light illumination at 2.0 mW/cm^2^ for 30, 60, 90, and 120 min. Data are represented by mean ± standard deviation, where *n* = 3. Significant differences (*p* < 0.05) between each treatment are indicated by different letters above the bar.

The O_2_•^−^ generation from the intermediates during the photolysis of TC under blue light illumination was investigated. The O_2_•^−^ was analysed by the NBT reduction method in this study. The effects of the illumination time of blue light on the NBT reduction during the photolysis of TC is shown in Figure 5. The photochemical reaction of TC increased with the reaction time under blue light illumination, confirming that O_2_•^−^ could be generated from TC photolysis with blue light illumination in an alkaline solution.

### 3.4. Effects of TC Treated With Blue Light Illumination on E. coli. Viability 

**Figure 6:**
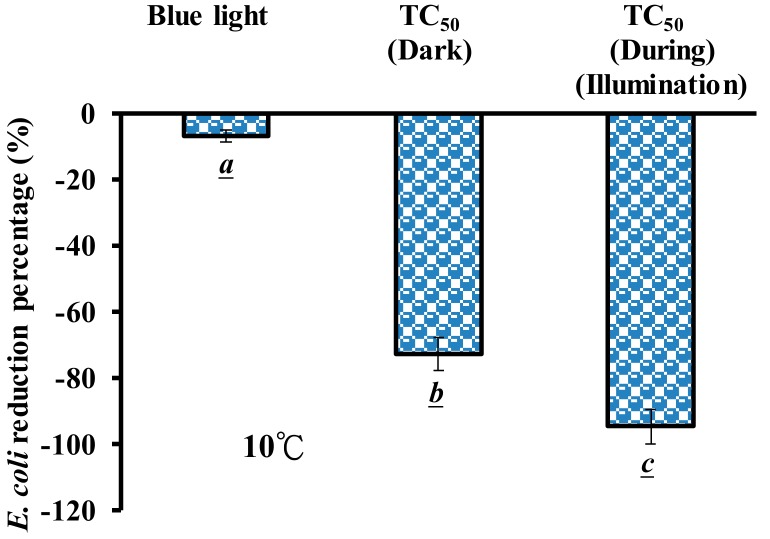
Effects of TC treated with blue light illumination on the viability of *E. coli* at 10 ± 1 °C. As a control, 50 mg/L TC in the dark was used. The reduction of *E. coli* during the photochemical reaction process was treated with 50 mg/L TC under blue light illumination at 2.0 mW/cm^2^ for 120 min (during illumination treatment). Data are represented by mean ± SD, where *n* = 3. Statistical differences (*p* < 0.05) between groups are indicated by the different letters below each bar.

The effects of TC treated with blue light illumination (during illumination treatment) on *E. coli* survivability were observed. The reduction percentage of *E. coli* increased with the addition of TC, as shown in Figure 6. According to Figure 6, blue light (2.0 mW/cm^2^ for 120 min) alone inactivated 6.9% *E. coli.* A 72.8% inactivation rate was attained with 50 mg/L TC in the dark for 120 min at 10 ± 1 °C. We found no significant difference (*p* = 0.066) in the inactivation rate of *E. coli* by PPT (50 mg/L TC after 2.0 mW/cm^2^ blue light illumination for 120 min) or the presence of 50 mg/L TC in the dark (data not shown). However, a 94.7% inactivation rate of *E. coli* at 10 ± 1 °C was attained with 50 mg/L TC treated with blue light illumination at 2.0 mW/cm^2^ for 120 min (during illumination treatment), as shown in Figure 6. TC treated with blue light illumination could increase the inactivation rate of *E. coli*. As shown in Figure 5, O_2_•^−^ could be generated from TC photolysis in an alkaline solution. Under specific circumstances, the O_2_•^−^ generated from TC under blue light illumination can enhance the inactivation rate of *E. coli*.

### 3.5. Effects of the Temperature of PPT and TC During Blue Light Illumination on MDR E. coli Viability

**Figure 7:**
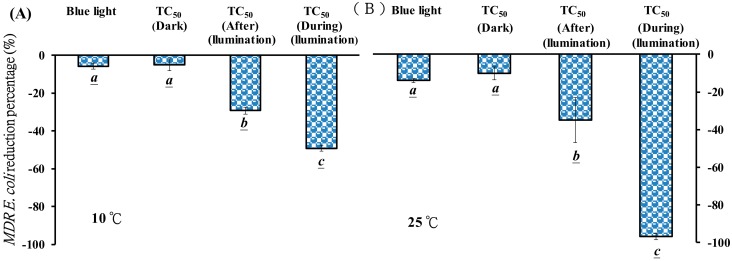
(**A**) Effects of photolysis product of TC (PPT) and TC under blue light illumination treatment on *MDR E. coli* viability at 10 ± 1 °C. (**B**) Effects of PPT and TC, under blue light illumination treatment, on *MDR E. coli* viability, with 25 ± 3 °C. 50 mg/L TC in the dark used as a control. The reduction of *MDR E. coli* was treated with PPT (after illumination treatment) and with a photochemical reaction process treated with 50 mg/L TC under blue light illumination at 2.0 mW/cm^2^ for 120 min (during illumination treatment). Data are represented by mean ± SD, where *n* = 3. Statistical differences (*p* < 0.05) between the groups are indicated by the different letters below each bar.

The effects of PPT and TC under blue light illumination treatment on the *MDR E. coli* survivability were observed at different temperatures. As shown in Figure 7A, a blue light illumination alone, at 2.0 mW/cm^2^ for 120 min, induced 5.7% inactivation of *MDR E. coli*, and a 4.9% inactivation rate was attained with 50 mg/L TC treatment for 120 min in the dark at 10 ± 1 °C. However, an inactivation rate of almost 50% of *MDR E. coli* was attained with 50 mg/L TC treated with blue light illumination at 2.0 mW/cm^2^ for 120 min (during illumination treatment), as shown in Figure 7A. 

This study investigated the effects of PPT and TC under blue light illumination treatment on the viability of *MDR E. coli* at room temperature (25 ± 3 °C). As shown in Figure 7B, the reduction percentage of *MDR E. coli* increased at room temperature. The temperature of the system was kept at 25 ± 3 °C, and the inactivation rate of *MDR E. coli* was increased to 96.6% with 50 mg/L TC under blue light illumination (during illumination treatment).

## 4. Discussion

To the best of our knowledge, we report the first successful photodynamic inhibition of *MDR E. coli* by photolysis TC with 462 nm blue light. Compared to the green and red light, blue light excitation provided the highest efficacy of TC degradation. To decompose TC from water, a heavy metal-based catalyst, such as TiO_2_ [35,36], SrTiO_3_ [37], ZnO [38], Fe_2_O_3_ [39], and Fe^2+^ or Fe^3+^/H_2_O_2_ [40], are usually added and activated with UV radiation. However, heavy metals and UV radiation are hazardous to the environment and animals. Blue light irradiation is completely safe for animals and the environment. The emission spectra of blue LED light is always pure, clear, and in a narrow wavelength range. TC is susceptible to blue light and O_2_•^−^ generated by the photosensitized oxidation of TC under blue light illumination, as shown in Figure 5. The ROS from TC photolysis may be important for predicting the kinetics of TC degradation [19].

In TC at pH 7.8, two peaks at 272 nm and 364 nm were found in the spectrum of the dark control, as shown in Figure 3. TC is a linear fused tetracyclic nucleus, which possesses a benzene ring and has a strong UV absorption capacity. TC is unstable under UV illumination in an aqueous solution [41] and photodegradation occurs as a consequence of the ROS generation from the photoreaction of TC via the excited states of the oxygen-containing species and the self-sensitization of TC [19]. Light with a shorter wavelength, such as UV light, has a high energy and can be strongly absorbed by TC to initiate a photochemical reaction. 

Chen et al. reported that O_2_•^−^ occurs in TC aqueous solution under simulated sunlight illumination [19]. The O_2_•^−^ can disproportionally generate H_2_O_2_, which can be decomposed to •OH under light illumination [42]. Liu et al. investigated the •OH occurring in oxytetracycline (OTC) aqueous solution under UV light illumination and found that the oxygen molecule could accept an electron to form O_2_•^−^, be decomposed to yield H_2_O_2_ [43] and, in turn, yield oxygen-dependent •OH under light illumination. The hydroxylation byproducts and water loss from the OTC photoreaction could be generated in OTC aqueous solution under UV light illumination [43]. The production of OH radicals from the photolysis of the –O–O– peroxidic bond via H_2_O_2_ under UV illumination can attack organic molecules [42]. Niu et al. reported that the TC photoreaction can react with O_2_•^−^ directly and undergo O_2_•^−^-mediated self-sensitized photolysis [34]. 

As shown in Figure 2, TC is a phenolic compound. It has been reported that phenolic compounds are not stable and are easily dissociated at a phenolic proton to form a phenolate anion via an electron transfer mechanism in alkaline aqueous circumstances [44]. The increase in pH value is proposed as an indicator of local wound infection in second degree burn wounds. The pH rose (turned to alkaline) prior to the onset of clinical signs of local infection, which were reported in 6 cases compared to 20 noninfected cases [45]. TC was unstable under alkaline aqueous conditions with the illumination of blue light. As seen in Figure 5, the aerobic photo-oxidative processes of TC activated by blue light illumination generated abundant O_2_•^−^. Several possible major processes of TC photodegradation under blue light illumination are shown in equations (3)–(6).

(3) $$\mathrm{TC}+O_{2}+e^{-}\overset{h\nu }{\to }\;\mathrm{TC}{•}^{+}+\;O_{2}{•}^{-}$$

O_2_•^−^ + H^+^ + e^− ^**→** HOO• (4)

2HOO• + H_2_O **→** H_2_O_2_ + O_2_ (5)

(6) $$H_{2}O_{2}+e^{-}\overset{h\nu }{\to }\;2\;•\mathrm{OH}$$

**Figure 8:**
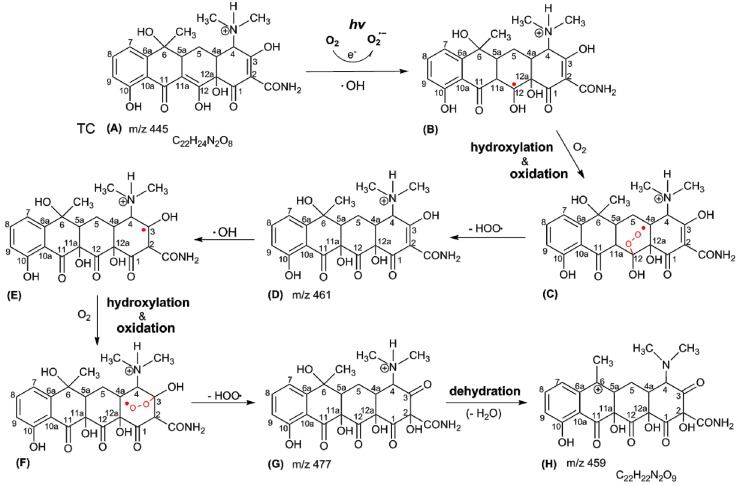
Proposed scheme for the TC photolysis.

The transformation products of TC are time-dependent evolution profiles during TC photodegradation [46]. Under alkaline aqueous conditions and blue light illumination, the mass spectrum of the photoreaction product of TC (PPT) had characteristic ion signals in m/z 459, 445, 249.1 Da, as shown in Figure 4E. Based on these results, we propose a TC photoreaction mechanism under blue light illumination, including hydroxylation, secondary alcohol oxidation, and dehydration, as shown in Figure 8.

As shown in Figure 5 and Equations (3)–(6), O_2_•^−^ and •OH could be generated by the photosensitized oxidation of TC under blue light illumination. Generally, as an electrophile, •OH can react mainly through a hydrogen abstraction reaction, hydroxyl addition to a double bond and an electron transfer [43]. The TC has the molecular formula of C_22_H_24_N_2_O_8_, as shown in Figure 8A. The hydroxylation of TC is an important reaction process. The ketone/enol sites of C2–C3 and C11a–C12 are sensitive to the OH reaction, caused by a hydroxyl addition to a double bond [47], and are most likely to be attacked by •OH. The double bond on C11a–C12 and C2–C3 can be broken by an •OH attack when the oxidation of TC and the hydroxyl group (–OH) is added to the carbon at C11a and C2. The hydroxyl group of C12 is converted into a ketone group (=O), and a compound D (m/z 461) is generated via the oxidized C12, as shown in Figure 8 (D). In addition to further hydroxylation and oxidation, the compound G (m/z 477) could be generated via the oxidation of C3, as shown in Figure 8G [48]. Finally, the compound H (m/z 459) is produced via the dehydration of the hydroxyl group at C6 on the compound G, as shown in Figure 8H [49]. The photoreaction product of TC has the molecular formula C22H22N2O9 and an exact mass of 458.44 g/mol.

Liang et al. reported that riboflavin or FMN treated with blue light illumination produced a large amount of O_2_•^−^ that degraded crystal violet [14] and led to the inactivation of *E. coli* and MRSA through ROS formation [3,13,30]. It has been reported that catechin hydrates under blue light illumination, and O_2_•^−^ was generated from photo-oxidation, increasing the inactivation rate of *A. baumannii*, including the carbapenem-resistant, *A. baumannii* [12,50]. The O_2_•^−^ generation in cells may induce DNA degradation, membrane peroxidation, and the destruction of endothelial cells [51]. We found in the present study that blue light is also very effective in photolysis TC and produced an ample amount of O_2_•^−^ to inactivate *E. coli and MDR E coli.* TC without light exposure had negligible effects on *MDR E. coli* survival, as shown in Figure 7A, which may be attributed to the drug efflux pumps existing in *MDR E. coli* that can pump drugs into the extracellular space [52,53]. The photolysis product of TC (PPT) significantly inactivated *MDR E. coli.* PPT without light exposure can inactivate 30% of *MDR E. coli*. The conformational changes of TC may affect the chemical milieu in the solution. With blue light excitation at a cold temperature or at room temperature, the inactivation of *MDR E coli* increased with temperature, suggesting that PPT may be an intermediate and unstable product.

The *LD*_50_ of *MDR E. coli* was used as a bacterial inactivation test and indicated the efficiency of TC photolysis. A 49% inactivation rate of *MDR E. coli* was attained with TC during blue light illumination at 10 ± 1 °C, as shown in Figure 7A. This inactivation rate is equivalent to a bactericidal agent, since it inhibits bacterial growth by at least 3 logs. It is possible to increase the bactericidal effects by increasing TC concentrations and/or light doses. By simply increasing the temperature from 10 °C to 25 °C during light exposure, a higher degree of TC photolysis and more O_2_•^−^ was formed, leading to a 96.6% *MDR E. coli* inactivation. The viable colony of the surviving *MDR E. coli* were below 8 CFU/plate, which indicated a 4–5 log growth inhibition. Blue light can be considered a new illumination source for the TC photoinactivation of *MDR E. coli*. 

## 5. Conclusions

The study revealed the significant inhibition of *MDR E coli* by shining blue light on a medium containing TC at 10 °C. The inhibition can be increased by 1 to 2 logs simply by photolysis TC at 25 °C, with the same light dose and drug concentration. Chromatographic and mass spectrometry analyses showed that the photodynamic effects yield highly reactive O_2_•^−^ to inactivate the bacteria. This photodynamic therapy may have potential as a new approach to the treatment of surface skin infections or deep subcutaneous or muscular infections by inserting a fiber optic to deliver blue light to the affected tissues.

## References

[1] Esposito S., Bassetti M., Concia E., De Simone G., De Rosa F.G., Grossi P., Novelli A., Menichetti F., Petrosillo N., Tinelli M. (2017). Diagnosis and management of skin and soft-tissue infections (SSTI). A literature review and consensus statement: An update. J. Chemother..

[2] Cardona A.F., Wilson S.E. (2015). Skin and soft-tissue infections: A critical review and the role of telavancin in their treatment. Clin. Infect. Dis..

[3] Liang J.Y., Cheng C.W., Yu C.H., Chen L.Y. (2015). Investigations of blue light-induced reactive oxygen species from flavin mononucleotide on inactivation of *E. coli*. J. Photochem. Photobiol. B Biol..

[4] Tadesse D.A., Zhao S., Tong E., Ayers S., Singh A., Bartholomew M.J., McDermott P.F. (2012). Antimicrobial drug resistance in *Escherichia coli* from humans and food animals, United States, 1950–2002. Emerg. Infect. Dis..

[5] Von Baum H., Marre R. (2005). Antimicrobial resistance of *Escherichia coli* and therapeutic implications. Int. J. Med. Microbiol..

[6] Chopra I., Roberts M. (2001). Tetracycline antibiotics: Mode of action, applications, molecular biology, and epidemiology of bacterial resistance. Microbiol. Mol. Biol. Rev..

[7] Roberts M.C. (1996). Tetracycline resistance determinants: Mechanisms of action, regulation of expression, genetic mobility, and distribution. FEMS Microbiol. Rev..

[8] McEwen S.A., Fedorka-Cray P.J. (2002). Antimicrobial use and resistance in animals. Clinical infectious diseases: An official publication of the Infectious. Diseases Soc. Am..

[9] Daghrir R., Drogui P. (2013). Tetracycline antibiotics in the environment: A review. Environ. Chem. Lett..

[10] Fairbrother J.M., Nadeau E. (2006). *Escherichia coli*: On-farm contamination of animals. Rev-Off. Int. Epizoot..

[11] Nhung N.T., Thuy C.T., Trung N.V., Campbell J., Baker S., Thwaites G., Hoa N.T., Carrique-Mas J. (2015). Induction of antimicrobial resistance in *Escherichia coli* and non-typhoidal salmonella strains after adaptation to disinfectant commonly used on farms in Vietnam. Antibiotics.

[12] Yang M.J., Hung Y.A., Wong T.W., Lee N.Y., Yuann J.M., Huang S.T., Wu C.Y., Chen I.Z., Liang J.Y. (2018). Effects of blue-light-induced free radical formation from catechin hydrate on the inactivation of *Acinetobacter baumannii*, including a carbapenem-resistant strain. Molecules.

[13] Liang J.Y., Yuann J.M., Cheng C.W., Jian H.L., Lin C.C., Chen L.Y. (2013). Blue light induced free radicals from riboflavin on *E. coli* DNA damage. J. Photochem. Photobiol. B Biol..

[14] Liang J.Y., Yuann J.P., Hsie Z.J., Huang S.T., Chen C.C. (2017). Blue light induced free radicals from riboflavin in degradation of crystal violet by microbial viability evaluation. J. Photochem. Photobiol. B Biol..

[15] Halliwell B., Gutteridge J.M. (1990). Role of free radicals and catalytic metal ions in human disease: An overview. Meth. Enzymol..

[16] Juen J.W., Jian H.L., Liang J.Y. (2010). The effect of illuminance on light induced reduction of nitro blue tetrazolium. MC-Trans. Biotechnol..

[17] Hasan T., Kochevar I.E., McAuliffe D.J., Cooperman B.S., Abdulah D. (1984). Mechanism of tetracycline phototoxicity. J. Investig. Dermatol..

[18] Redelsperger I.M., Taldone T., Riedel E.R., Lepherd M.L., Lipman N.S., Wolf F.R. (2016). Stability of doxycycline in feed and water and minimal effective doses in tetracycline-inducible systems. J. Am. Assoc. Lab. Anim. Sci..

[19] Chen Y., Hu C., Qu J., Yang M. (2008). Photodegradation of tetracycline and formation of reactive oxygen species in aqueous tetracycline solution under simulated sunlight irradiation. J. Photochem. Photobiol. A Chem..

[20] Andreozzi R., Raffaele M., Nicklas P. (2003). Pharmaceuticals in STP effluents and their solar photodegradation in aquatic environment. Chemosphere.

[21] Jiao S., Zheng S., Yin D., Wang L., Chen L. (2008). Aqueous photolysis of tetracycline and toxicity of photolytic products to luminescent bacteria. Chemosphere.

[22] Tim M. (2015). Strategies to optimize photosensitizers for photodynamic inactivation of bacteria. J. Photochem. Photobiol. B Biol..

[23] Hamblin M.R. (2016). Antimicrobial photodynamic inactivation: A bright new technique to kill resistant microbes. Curr. Opin. Microbiol..

[24] Wong T.W., Wu E.C., Ko W.C., Lee C.C., Hor L.I., Huang I.H. (2017). Photodynamic inactivation of methicillin-resistant *Staphylococcus aureus* by indocyanine green and near infrared light. Dermatol. Sin..

[25] Maisch T., Hackbarth S., Regensburger J., Felgentrager A., Baumler W., Landthaler M., Roder B. (2011). Photodynamic inactivation of multi-resistant bacteria (PIB)—A new approach to treat superficial infections in the 21st century. J. Dtsch. Dermatol. Ges..

[26] He Y., Huang Y.Y., Xi L., Gelfand J.A., Hamblin M.R. (2018). Tetracyclines function as dual-action light-activated antibiotics. PLoS ONE.

[27] Martin J.P., Colina K., Logsdon N. (1987). Role of oxygen radicals in the phototoxicity of tetracyclines toward *Escherichia coli* B. J. Bacteriol..

[28] Chen Y., Li H., Wang Z., Tao T., Wei D., Hu C. (2012). Photolysis of chlortetracycline in aqueous solution: Kinetics, toxicity and products. J. Environ. Sci..

[29] Huang J.J., Hu H.Y., Wu Y.H., Wei B., Lu Y. (2013). Effect of chlorination and ultraviolet disinfection on tetA-mediated tetracycline resistance of *Escherichia coli*. Chemosphere.

[30] Wong T.W., Cheng C.W., Hsieh Z.J., Liang J.Y. (2017). Effects of blue or violet light on the inactivation of *Staphylococcus aureus* by riboflavin-5′-phosphate photolysis. J. Photochem. Photobiol. B Biol..

[31] Gavilan R.E., Nebot C., Veiga-Gomez M., Roca-Saavedra P., Vazquez Belda B., Franco C.M., Cepeda A. (2016). A Confirmatory Method Based on HPLC-MS/MS for the Detection and Quantification of Residue of Tetracyclines in Nonmedicated Feed. J. Anal. Methods Chem..

[32] Cheng C.W., Chen L.Y., Chou C.W., Liang J.Y. (2015). Investigations of riboflavin photolysis via coloured light in the nitro blue tetrazolium assay for superoxide dismutase activity. J. Photochem. Photobiol. B Biol..

[33] Russell L.V. (1990). Comprehensive review of vitamin B_2_ analytical methodology. J. Micronutr. Anal..

[34] Niu J., Li Y., Wang W. (2013). Light-source-dependent role of nitrate and humic acid in tetracycline photolysis: Kinetics and mechanism. Chemosphere.

[35] Bouafıa-Cherguı S., Zemmourı H., Chabanı M., Bensmaılı A. (2016). TiO_2_-photocatalyzed degradation of tetracycline: Kinetic study, adsorption isotherms, mineralization and toxicity reduction. Desalin. Water Treat..

[36] Reyes C., Fernandez J., Freer J., Mondaca M., Zaror C., Malato S., Mansilla H. (2006). Degradation and inactivation of tetracycline by TiO_2_ photocatalysis. J. Photochem. Photobiol. A Chem..

[37] Cai F., Tang Y., Chen F., Yan Y., Shi W. (2015). Enhanced visible-light-driven photocatalytic degradation of tetracycline by Cr^3+^ doping SrTiO_3_ cubic nanoparticles. RSC Adv..

[38] Wang H., Yao H., Pei J., Liu F., Li D. (2016). Photodegradation of tetracycline antibiotics in aqueous solution by UV/ZnO. Desalin. Water Treat..

[39] Saghi M., Mahanpoor K. (2017). Photocatalytic degradation of tetracycline aqueous solutions by nanospherical α-Fe_2_O_3_ supported on 12-tungstosilicic acid as catalyst: Using full factorial experimental design. Int. J. Ind. Chem..

[40] Yamal-Turbay E., Jaén E., Graells M., Pérez-Moya M. (2013). Enhanced photo-Fenton process for tetracycline degradation using efficient hydrogen peroxide dosage. J. Photochem. Photobiol. A Chem..

[41] Davies A.K., McKellar J.F., Phillips G.O., Reid A.G. (1979). Photochemical oxidation of tetracycline in aqueous solution. J. Chem. Soc. Perkin Trans..

[42] Li C., Gao N., Wang L., Shen Y. (2012). Hydrogen peroxide-assisted low pressure UV photodegradation of atrazine in aqueous solution. Int. J. Environ. Stud..

[43] Liu Y., He X., Fu Y., Dionysiou D.D. (2016). Degradation kinetics and mechanism of oxytetracycline by hydroxyl radical-based advanced oxidation processes. Chem. Eng. J..

[44] Chen L.Y., Cheng C.W., Liang J.Y. (2015). Effect of esterification condensation on the Folin-Ciocalteu method for the quantitative measurement of total phenols. Food Chem..

[45] Ono S., Imai R., Ida Y., Shibata D., Komiya T., Matsumura H. (2015). Increased wound pH as an indicator of local wound infection in second degree burns. Burns.

[46] Li S., Hu J. (2016). Photolytic and photocatalytic degradation of tetracycline: Effect of humic acid on degradation kinetics and mechanisms. J. Hazard. Mater..

[47] Broszkiewicz R., Söylemez T., Schulte-Frohlinde D. (1982). Reactions of OH radicals with acetylacetone in aqueous solution. A pulse radiolysis and electron spin resonance study. Z. Naturforsch. B J. Chem. Sci..

[48] Dalmazio I., Almeida M.O., Augusti R., Alves T.M. (2007). Monitoring the degradation of tetracycline by ozone in aqueous medium via atmospheric pressure ionization mass spectrometry. J. Am. Soc. Mass Spectrom..

[49] Wang Y., Zhang H., Chen L. (2011). Ultrasound enhanced catalytic ozonation of tetracycline in a rectangular air-lift reactor. Catal. Today.

[50] Liang J.Y., Wu J.Y., Yang M.Y., Hu A., Chen L.Y. (2016). Photo-catalytic polymerization of catechin molecules in alkaline aqueous. J. Photochem. Photobiol. B Biol..

[51] Brown R.K., McBurney A., Lunec J., Kelly F.J. (1995). Oxidative damage to DNA in patients with cystic fibrosis. Free Radic. Biol. Med..

[52] Heng J., Zhao Y., Liu M., Liu Y., Fan J., Wang X., Zhao Y., Zhang X.C. (2015). Substrate-bound structure of the *E. coli* multidrug resistance transporter MdfA. Cell Res..

[53] Putman M., van Veen H.W., Konings W.N. (2000). Molecular properties of bacterial multidrug transporters. Microbiol. Mol. Biol. Rev..

